# An N-ethyl-N-Nitrosourea Mutagenesis Screen in Mice Reveals a Mutation in *Nuclear Respiratory Factor 1* (*Nrf1)* Altering the DNA Methylation State and Correct Embryonic Development

**DOI:** 10.3390/ani11072103

**Published:** 2021-07-15

**Authors:** Maria Alba Sorolla, Marta Marqués, Eva Parisi, Anabel Sorolla

**Affiliations:** 1 Research Group of Cancer Biomarkers, Lleida Institute for Biomedical Research Dr. Pifarré Foundation IRBLleida, 25198 Lleida, Spain; msorolla@irblleida.cat (M.A.S.); eparisi@irblleida.cat (E.P.); 2 Department of Medicine, University of Lleida, 25198 Lleida, Spain; mms30@alumnes.udl.cat; 3 Epigenetics Laboratory, QIMR Berghofer Medical Research Institute, Herston, Brisbane, QLD 4006, Australia

**Keywords:** NRF1, Nrf1, ENU mutagenesis, epigenetics, transcription factor, embryonic development, DNA methylation

## Abstract

**Simple Summary:**

In this work, we aimed to discover unknown genes that are important in the regulation of other genes. These genes often play an important role during the development of the embryo. By screening thousands of mice, we found a gene, namely, *Nuclear Respiratory Factor 1* (*Nrf1*), that controls the switching on and off of other genes. Mice with a defective *Nrf1* present lesser levels of the gene and embryonic delay. When the mutation is in both chains of the DNA, mice are not born and die in the uterus. Our work unveils a novel, previously unknown functionality of *Nrf1* and provides a new mice model for the study of diseases caused by a defective *Nrf1*.

**Abstract:**

We have established a genome-wide N-ethyl-N-nitrosourea (ENU) mutagenesis screen to identify novel genes playing a role in epigenetic regulation in mammals. We hypothesize that the ENU mutagenesis screen will lead to the discovery of unknown genes responsible of the maintenance of the epigenetic state as the genes found are modifiers of variegation of the transgene green fluorescent protein (GFP) expression in erythrocytes, which are named *MommeD*. Here we report the generation of a novel mutant mouse line, *MommeD46*, that carries a new missense mutation producing an amino acid transversion (L71P) in the dimerization domain of Nuclear Respiratory Factor 1 (Nrf1). The molecular characterization of the mutation reveals a decrease in the *Nrf1* mRNA levels and a novel role of Nrf1 in the maintenance of the DNA hypomethylation in vivo. The heritability of the mutation is consistent with paternal imprinting and haploinsufficiency. Homozygous mutants display embryonic lethality at 14.5 days post-coitum and developmental delay. This work adds a new epi-regulatory role to Nrf1 and uncovers unknown phenotypical defects of the *Nrf1* hypomorph. The generated mouse line represents a valuable resource for studying NRF1-related diseases.

## 1. Introduction

Mutagenesis screens for the identification of modifiers of position-effect variegation (PEV) in Drosophila melanogaster have been pivotal for the development of the field of epigenetics [1]. These screens, performed in X-ray irradiated flies, have identified PEV alleles able to modify the expression of various genes presenting variegated expression, including the white (w) locus which leads to an easily-identifiable mosaicism of white and red patches in the eyes [1]. The identified alleles have been classified as dominant enhancers or suppressors of variegation, namely E(var)s or Su(var)s [2,3], depending on whether they enhance or suppress heterochromatin-mediated gene silencing. Similarly, mutagenesis screens have been performed in mammals for the identification of PEV modifiers. For this purpose, our group has established an N-ethyl-N-nitrosourea (ENU) forward dominant mutagenesis screen in transgenic mice (Line3) expressing green fluorescent protein (GFP) in erythrocytes in a variegated manner [4,5]. The transgene is composed by the human α1-globin gene promoter and the human α-globin locus enhancer region (HS-40) which is linked to the human GFP (reporter gene) [5]. Such a construct is expressed in approximately 55% of the erythrocytes of Line3 mice [6]. In this screen, enhancers and suppressors of variegation are easily identified by flow cytometry with a drop of blood [7,8], and are denominated as *MommeDs* (modifiers of murine metastable epialleles, Dominant). In our laboratory, this screen has successfully identified more than 40 unknown and known genes playing a role in fundamental epigenetic processes in mammals [4,6,7,8,9,10,11,12,13,14]. Nearly all these genes are also required for correct embryonic development [6,7,8,10,13] and have been linked to a broad range of syndromes and diseases. For instance, reduced levels of Structural Maintenance of Chromosomes Flexible Hinge Domain Containing 1 (SMCHD1) have been associated to the human facioscapulohumeral dystrophy type 2 and cancer [15,16], a loss-of-function mutation in Wiz has been associated with anxiety-like behavior in mice [14] and loss of Rearranged L-Myc fusion (RLF) has been shown to cause heart defects in mice [17].

Herein, we report the identification and characterization of a novel mutation in the Nuclear Respiratory Factor 1 gene (*Nrf1*). The mice line is named *MommeD46*. NRF1 is a transcription factor initially found to bind the cytochrome c promoter and activate transcription [18]. NRF1 regulates respiration, mitochondrial biogenesis and mitochondrial DNA transcription and replication [19,20,21]. Consistent with being identified in our mutagenesis screen, Nrf1 plays a role in the maintenance of DNA hypomethylation, because the under-functioning Nrf1 induces hypermethylation. Moreover, the novel *Nrf1* mutation is homozygous lethal and presents a characteristic hereditability consistent with paternal imprinting and haploinsufficiency. This work adds a new epi-regulatory role to Nrf1 and discovers unknown embryonic effects of a novel *Nrf1* hypomorph. The generated mouse line could be used for modeling and studying diseases caused by an underfunctioning NRF1.

## 2. Materials and Methods

### 2.1. Animals

**Figure 1:**
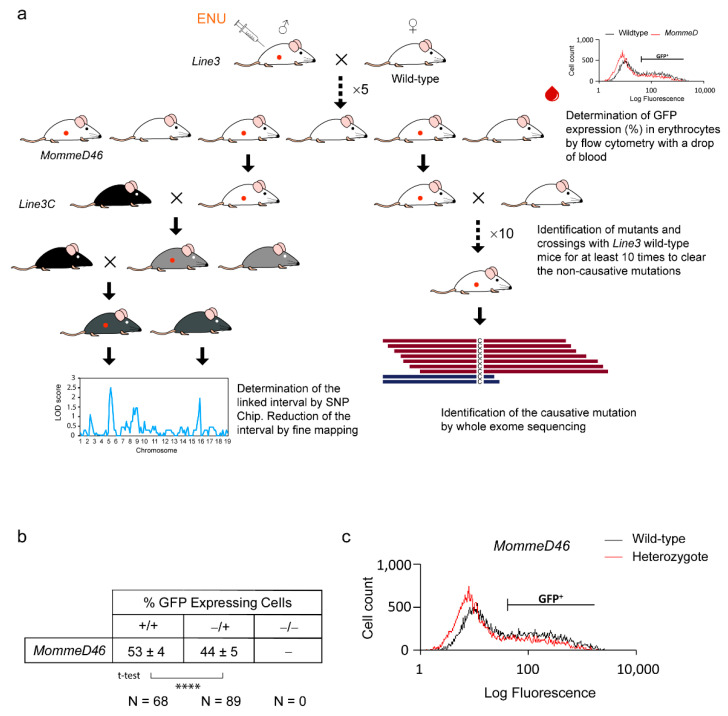
*MommeD46* is an enhancer of variegation. (**a**) Schematic representation of the ENU mutagenesis gene discovery pipeline. (**b**) Table indicating the average percentage of GFP positive erythrocytes ± standard deviation (SD) for *Nrf1^+/+^* and *Nrf1^MommeD46/+^*. *Nrf1^MommeD46/MommeD46^* were not viable at weaning. (**c**) Graph of the GFP fluorescence profile obtained by flow cytometry from a drop of blood of *Nrf1^+/+^* (wild-type) and *Nrf1^MommeD46/+^* (heterozygote). Statistical significance was determined by two-tailed Student’s t-test, **** indicates *p* < 0.00005.

All animal experiments were performed in accordance with protocols approved by the Animal Ethics Committee of QIMR Berghofer Medical Research Institute. All the animal experiments comply with the ARRIVE guidelines. Pups were weaned at three-weeks-old and bred when males and females were eight and six-weeks-old, respectively. The ENU screen was performed in FVB/NJ inbred transgenic mice, namely Line3, homozygous for the green fluorescence protein (GFP) transgene under the control of the α-globin promoter, as previously described [4]. *MommeD46* was maintained in this mice line and crossed for at least five generations with wild-type FVB/NJ mice, down from the mutant founder. Line3C mice were used for the linkage analysis (Figure 1a). Line3C was obtained by crossing Line3 with mice of strand C57BL/6J for ten generations and selecting for the offspring carrying the GFP transgene by flow cytometry. C57BL/6J mice were purchased from the Animal Resources Centre (Perth, WA, Australia). Sperm of *MommeD46* is cryopreserved at the Australian Phenome Bank at the Australian National University (2601 ACT, Australia).

### 2.2. Embryo Dissections

Heterozygous *MommeD46* mice were crossed and females were daily monitored for vaginal plugs. A vaginal plug was counted as 0.5 days post-coitum (dpc). Pregnant females were sacrificed by CO_2_ inhalation, dissected and embryos isolated and visualized under a binocular dissecting microscope.

#### Determination of GFP Expression in Erythrocytes

A tiny cut in the tail tip was performed in manually strained (three-weeks-old) mice to extract a drop of blood of three-weeks-old mice to be then dissolved in Osmosol buffer and analyzed using the benchtop flow cytometer Guava easyCyte HT (Merck/Millipore, Darmstadt, Germany). A gating was set to exclude 99.9% of erythrocytes not expressing the GFP.

### 2.3. Linkage Analysis by SNP Chip

**Figure 2:**
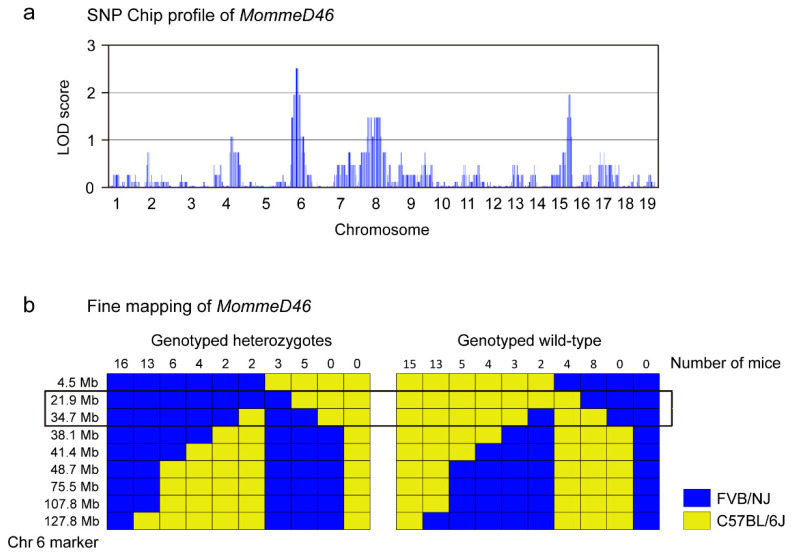
The point mutation in the *MommeD46* is localized in Chromosome 6. (**a**) Manhattan plot showing the linked intervals identified by SNP Chip in *MommeD46*. The y-axis represents the logarithm of the odds (LOD) score and the x-axis represents the Chr number. (**b**) Scheme of the fine mapping performed in the linked interval identified by SNP Chip in Chr 6 comprising a genomic region ranging from 4.5 Mb to 127.8 Mb. The numbers indicate the number of mice having a heterozygous or wild-type genotype in the genomic regions indicated. The blue boxes represent the existence of FVB/NJ-specific SNPs and the yellow boxes the existence of C57BL/6J-specific SNPs at the given markers.

DNA from tail tips from 15 mice phenotypically expressing GFP in 55% of the erythrocytes (wild-type) and 15 expressing lower GFP percentages (*MommeD46*) from the backcross between heterozygous *MommeD46* with Line3 background (FVB/NJ strain) and Line3C (C57BL/6J strain) mice were included in the linkage analysis (Figure 1a). Mice were at least three-weeks-old. The Mouse Medium Density Linkage Panel from Illumina (San Diego, CA, USA) was used for single nucleotide polymorphism (SNP) genotyping. The panel contains 766 measurable differential SNPs between C57BL/6J and FVB/NJ strains. Wild-type mice for the mutation should only have C57BL/6J-specific SNPs in the linked region while heterozygous *MommeD46* mice can have both C57BL/6J and FVB/NJ-specific SNPs. SNP Chip was performed according to Illumina instructions and SNP calling was carried out using the Genotyping module of the GenomeStudio v1.1 software (Illumina). Only call rates higher than 95 were accepted. Logarithm of odds (LOD) scores across the entire mouse genome were calculated and linked intervals identified (LOD scores ˃ 2, Figure 1a and Figure 2). Further fine mapping using additional SNP markers (Figure S1) was performed to reduce the linked interval.

### 2.4. Whole Exome Sequencing

Whole exome sequencing for the identification of the underlying mutation in *MommeD46* was carried out as previously described [7].

### 2.5. Methylation Analysis in the Transgene HS-40 Enhancer

DNA samples from three wild-type, three heterozygous and three homozygous *MommeD46* 9.5 dpc embryos were converted with bisulfite using the EpiTect Bisulfite Kit (Qiagen, Doncaster, VIC, Australia) in accordance with the manufacturer’s instructions. Bisulfite-converted DNA was amplified (first PCR) using specific oligonucleotides (Integrated DNA Technologies, IA, USA) for the HS-40 enhancer region, as follows: 5′-AAAATAAAATTTTTGGATTGTTATTATTATAA-3′ (Forward 1), 5′-ATATTTGTAATTTTAGTATTTTGGGAGGTT-3′ (Forward 2) and 5′-AATCTCTACTCACTACAAACTCCATCTC-3′ (Reverse) with the following cycling conditions: one cycle at 94 °C for 2 min; 35 cycles at 94 °C for 30 s, 60 °C for 30 s, 72 °C for 45 s; one cycle at 72 °C for 6 min. Next, a semi-nested PCR was performed with the first PCR product using the oligonucleotides Forward 2 and Reverse. The product was then ligated into a pGEM^®^-T Vector (Promega, Madison, WI, USA) as per Promega instructions. DH5α competent E. coli were transformed using the heat shock method and plated in LB agar plates containing ampicillin, x-gal and IPTG. Positive colonies (white) were picked and DNA was amplified to determine transgene insertion using the following oligonucleotides: 5′-ATTTAGGTGACACTATAG-3′ (Forward, SP6) and 5′-TAATACGACTCACTATAGGG-3′ (Reverse, T7) and PCR conditions according to the MangoTaq DNA polymerase protocol (Bioline Reagents Limited, London, UK). Plasmid DNA from positive colonies was then extracted with Qiagen Miniprep Kit (Germantown, MD, USA) and sequenced. The BIQ Analyser software was used to determine the quality of the conversion and for the analysis. Sequences with <98% of conversion rate and <80% of sequence matching as well as identical clones were excluded from the analysis. White and dark circles-containing diagrams, obtained from introducing the BIQ results’matrix to the software freshteapot, were used to represent the unmethylated and methylated CpG sites in the HS-40 enhancer, respectively.

### 2.6. Genotyping

**Figure 3:**
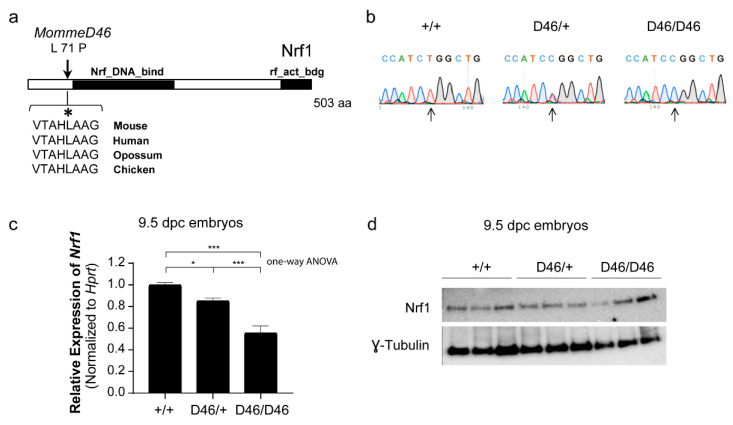
*MommeD46* harbors a point mutation in the third exon of *Nrf1* (**a**) Schematic representation of the mutation in the NRF1 protein and conservation of the indicated amino acid sequence across different species. (**b**) Representative Sanger sequences traces of amplicons derived from *Nrf1^+/−^*, *Nrf1^MommeD46/+^ and Nrf1^MommeD46/MommeD46^* 9.5 days post-coitum (dpc) embryos. (**c**) Graph of qRT-PCR relative expression values of *Nrf1* ± SD in *Nrf1^+/+^*, *Nrf1^MommeD46/+^ and Nrf1^MommeD46/MommeD46^* 9.5 dpc embryos. *Hprt* is used as normalizer. (**d**) Western Blot of Nrf1 using whole protein extracts from *Nrf1^+/+^*, *Nrf1^MommeD46/+^ and Nrf1^MommeD46/MommeD46^* 9.5 dpc embryos. γ-Tubulin is used as loading control. *D46* is an abbreviation of *MommeD46*. Statistical significance was determined by one-way ANOVA and post hoc Tukey test, * indicates *p* < 0.05 and *** indicates *p* < 0.0005.

Genotyping of *MommeD46* was performed by Sanger sequencing or by restriction enzyme digestion followed by gel electrophoresis. For both genotyping methods, DNA from tail tips (at least three-weeks-old) and embryos was extracted using a saturated salt solution containing 50 mM Tris pH 6.8, 20 mM EDTA pH 8, 2% SDS and 6.25 µg/mL proteinase K. DNA was precipitated with isopropanol and washed with 75% and 100% ethanol. DNA pellets were air dried and treated with 200 µL of RnaseA (10 mg/mL). Sanger sequencing was performed for 9.5 dpc embryos genotyping (Figure 3b). Wild-type, heterozygous and homozygous *MommeD46* embryos (Figure 3b) were homogenized. DNA was isolated and a genomic region of 321 base pairs (bp) covering the causative mutation in *Nrf1* was amplified by PCR using the oligonucleotides 5′-GGCTCAGCCAGTGTTTTCTTA-3′ (forward) and 5′-TTTGATCCCCAGCAGTGAA-3′ (reverse). The PCR product was then sequenced using the BigDye Terminator Sequencing Kit (Applied Biosystems, Foster City, CA, USA) and the previous forward oligonucleotide. The restriction enzyme digestion was performed for both adult and embryo genotyping. To do so, the same obtained above PCR product was subjected to digestion with HpaII (New England BioLabs, Ipswich, MA, USA). In wild-type *MommeD46* the enzyme did not cut, in heterozygous *MommeD46* the enzyme only cut one DNA strand, producing fragments of 321 bp, 173 bp and 148 bp, and in homozygous *MommeD46* the enzyme cut both DNA strands, producing fragments of 173 bp and 148 bp.

### 2.7. Determination of Nrf1 mRNA Levels by Quantitative Real-Time PCR

The determination of *Nrf1* mRNA levels was performed in three wild-type, three heterozygous and three homozygous *MommeD46* of at least three weeks of age, following a three-step process which included RNA isolation, cDNA conversion and quantitative real-time (qRT)-PCR. For RNA isolation, 9.5 dpc embryos were mechanically homogenized and treated with Trizol^®^ (Qiagen, Doncaster, VIC, Australia) and chloroform. RNA was precipitated with isopropanol and high salt solution (0.8 M NaCitrate and 1.2 M NaCl). RNA was washed with 75% ethanol, air dried and dissolved in DEPC water. RNA was converted into cDNA using SuperScriptIII reverse transcriptase and random hexamer primers (Invitrogen, Carlsbad, CA, USA). For the qRT-PCR, the forward oligonucleotide for *Nrf1* was designed at the boundary of the first exon and the second exon (5′-TGAGGTCGAATGGTATGTGGT-3′), and the reverse oligonucleotide 78 bp downstream (5′-ACACCCATGTTCATAGCAGCA-3′), leaving a product of 121 bp. The DNA amplification was performed using the Platinum SYBR Green qPCR Super Mix—UDG (Invitrogen, Carlsbad, CA, USA). The PCR was run in the Viia7 (Applied Biosystems, Mulgrave, VIC, Australia) and analyzed using the Viia7 software. *Hprt* was used as housekeeping gene, using the qRT-PCR oligonucleotides 5′-GGCCAGACTTTGTTGGATTT-3′ (forward) and 5′-ACTGGCAACATCAACAGGACT-3′ (reverse).

### 2.8. Determination of Nrf1 Protein Levels by Western Blot

Whole-protein extracts were prepared from three wild-type, three heterozygous and three homozygous *MommeD46* 9.5 dpc embryos. Embryo tissue was mechanically homogenized and lysed with lysis buffer containing urea 8 M, 1% sodium dodecyl sulfate, 10% glycerol, 0.5 mM dithiothreitol and 10 mM Tris pH 6.8, complemented with the serine protease inhibitor phenylmethylsulfonyl fluoride and half tablet of the protease inhibitor cocktail cOmplete^®^ from Roche (Swedesboro, NJ, USA) for 5 mL of lysis buffer. Next, samples were sonicated and quantified using the Pierce BCA Protein Assay Kit (ThermoFisher Scientific Australia Pty Ltd., Scoresby, VIC, Australia). Equal amounts of protein (30 µg) were loaded in a Mini-Protean precast 7.5% polyacrylamide gel from Bio-Rad (Hercules, CA, USA) and run at 100 V. Proteins were transferred into PVDF membranes following the wet transfer protocol, overnight at 4 °C at 20 V. Membranes were blocked with 5% of skim milk and 1% BSA in PBS-T. The anti-Nrf1 antibody from Novus Biologicals (Littleton, CO, USA) was incubated in the same solution overnight at 4 °C, 1:1000 dilution. A goat anti-rabbit secondary antibody conjugated to horseradish peroxidase (HRP) from DAKO, 1:5000 dilution in PBS-T, (Santa Clara, CA, USA) was added for one hour at room temperature. Clarity Western ECL substrate (Bio-Rad) was added in the membrane and the chemiluminescence signal was captured with the MF Chemi-Bis (DNR Bio-imaging systems). The γ-Tubulin antibody from Sigma-Aldrich (St Louis, MO, USA), 1:1000 dilution, was used as loading control.

### 2.9. Statistical Analysis

All the experiments were done in at least three biological replicates. Normality of data was assessed by Saphiro–Wilk or Kolmogorov–Smirnov tests, depending on the sample size. When the data were satisfied with normality assumption, statistical significance between mean differences of the groups was assessed by two-tailed Student’s t-test or one-way ANOVA and *p* < 0.05 was considered significant. Otherwise, the non-parametric Mann–Whitney U test was applied and *p* < 0.05 was considered significant. Chi–Square test (χ2) or Fisher’s exact test (F) were used to compare the observed proportion of genotypes with the expected Mendelian ratios when the number of events in a cell was ≥5 or <5, respectively, and a *p* value < 0.05 was deemed as significant. Levene’s test or F-test of equality of variances was used to determine variance differences between wild-type and heterozygous distributions, holding significance when *p* < 0.05. In this figure, two-way ANOVA was also employed. All analyses were performed using the software IBM SPSS Statistics version 24.0 (Chicago, IL, USA).

## 3. Results

### 3.1. MommeD46 Is an Enhancer of Variegation

The causative mutation in *MommeD46* induces a change in the GFP expression in erythrocytes. Wild-type *MommeD46* mice express the GFP in approximately 55% of red blood cells (Figure 1b), as previously shown for Line3 mice [7]. Heterozygous *MommeD46* mutants present 44 ± 5% of GFP-expressing erythrocytes, which is significantly less (*p* = 2.15 × 10^−28^) than the percentage observed in their wild-type counterparts (53 ± 4%). Figure 1c shows the flow cytometry GFP profiles of wild-type and heterozygous mutant *MommeD46*. Mutant mice show a lesser count. Therefore, *MommeD46* is an enhancer of variegation.

### 3.2. MommeD46 Carries a Point Mutation in the Nrf1 Gene

To elucidate the underlying mutation in *MommeD46*, we followed different strategies. First, we performed SNP Chip to determine the linked genomic interval where the mutation was located. We identified two linked regions with LOD score of 2 or higher in Chromosome (Chr) 6 and Chr 15 (Figure 2a). As the highest LOD score was in Chr 6, we narrowed down the linked interval in that Chr, comprising a region of 123.3 Mb, from the genomic location 4.5 Mb to 127.8 Mb (Figure S1). We exploited nine differential SNP markers between FVB/NJ and C57BL/6J at 4.5, 21.9, 34.7, 38.1, 41.4, 48.7, 75.5, 107.8, and 127.8 Mb. We found a number of supporting genotypically heterozygous mice, and thus, with C57BL/6J-specific SNPs, concentrated at 21.9 and 34.7 Mb. At the same locations, we also found genotypically wild-type mice (with FVB/NJ-specific SNPs) (Figure 2b). This observation indicated that the underlying mutation in *MommeD46* was located somewhere between 21.9 and 34.7 Mb. Later, we performed exome deep sequencing and we identified the causative mutation to be a missense mutation at the end of the third exon of *Nrf1* (Chr 6:30,040,135; USC Genome Browser) (Figure S2). This was confirmed by Sanger sequencing in 9.5 dpc embryos (Figure 3b). The mutation is a T ˃ C substitution that induces a Leucine (L) ˃ Proline (P) change at position 71 of the amino acid sequence of Nrf1, located before the predicted Nrf1 DNA binding domain. The affected amino acid is conserved between species (Figure 3a). The point mutation in *Nrf1* causes a decrease in the *Nrf1* mRNA levels as seen by qRT-PCR. In particular, *Nrf1^MommeD46/+^* and *Nrf1^MommeD46^*^/*MommeD46*^ 9.5 dpc embryos showed a 15% (*p* = 0.014) and a 44% reduction (*p* = 0.0004) in *Nrf1* mRNA levels, respectively, compared to *Nrf1*^+/+^ (Figure 3c). In contrast, the mutation did not induce a noticeable change in the Nrf1 protein levels as observed by Western blot (Figure 3d). In addition, the band quantification of the Western Blot did not show significant differences of band intensity between the groups when normalized to γ-Tubulin (Figure S3).

### 3.3. Nrf1 Mutants Display Enhanced DNA Methylation

**Figure 4:**
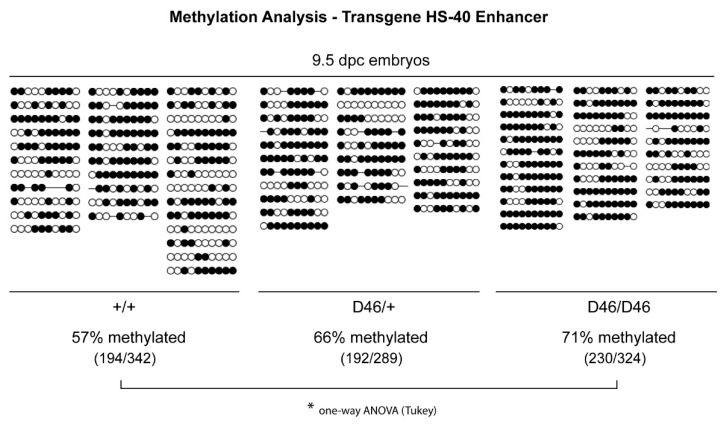
Nrf1 is involved in DNA methylation. Methylation of the CpG sites in the HS-40 enhancer region of the GFP transgene in *Nrf1^+/+^*, *Nrf1^MommeD46/+^ and Nrf1^MommeD46/MommeD46^* 9.5 dpc embryos. Three embryos per group were analyzed. Dark circles indicate methylated sites and white circles represent unmethylated sites. Statistical differences between the groups were determined by one-way ANOVA and post hoc Tukey test, * indicates *p* < 0.05.

To further prove a role of Nrf1 as epigenetic regulator in mammals, we performed bisulfite sequencing of the HS-40 enhancer region of the GFP transgene in wild-type, heterozygous and homozygous *MommeD46* 9.5 dpc embryos. We observed that *Nrf1*^+/+^ embryos presented 57% of the CpG islands methylated. This percentage increased up to 66% in *Nrf1^MommeD46^*^/+^ embryos, and up to 71% in *Nrf1^MommeD46^*^/*MommeD46*^ embryos (*p* = 0.049) (Figure 4). This result suggests that Nrf1 is involved in DNA methylation and a functional Nrf1 is essential to maintain a hypomethylated state, which is consistent with the fact that Nrf1 is an enhancer of variegation.

### 3.4. The Observed Hereditability of the Nrf1 Suggests Paternal Imprinting and Haploinsufficiency, and Normal Nrf1 Is Required for Normal Embryonic Development

**Figure 5:**
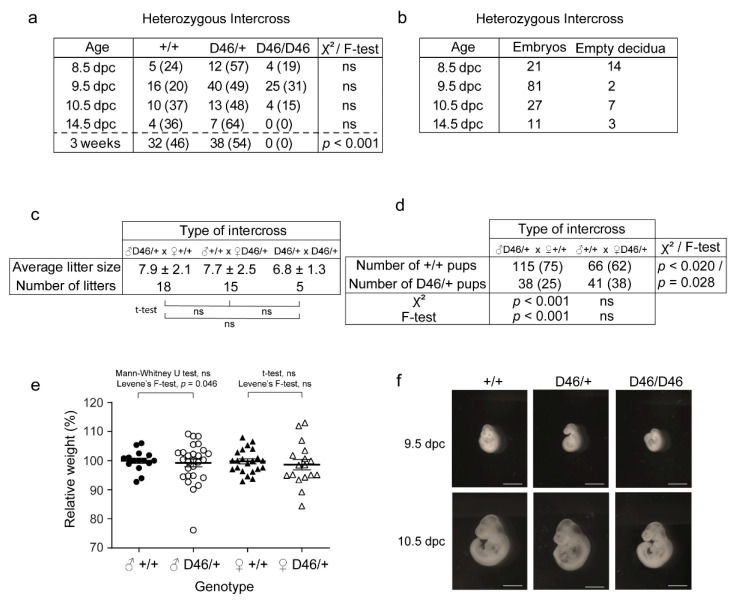
*MommeD46* shows developmental defects, reduced weight and non-Mendelian inheritance. (**a**) Table showing the number of *Nrf1^+/−^*, *Nrf1^MommeD46/+^ and Nrf1^MommeD46/MommeD46^* offspring at 8.5 dpc, 9.5 dpc, 10.5 dpc, 14.5 dpc and 3 weeks after birth obtained from the heterozygous intercross. The respective percentages are between parentheses. (**b**) Table showing the number of normal and abnormal (empty decidua) offspring at 8.5 dpc, 9.5 dpc, 10.5 dpc and 14.5 dpc obtained from the heterozygous intercross. (**c**) Table including the average litter size ± SD obtained from the intercrosses between male *Nrf1^MommeD46/+^* and female *Nrf1^+/+^*, male *Nrf1^+/+^* and female *Nrf1^MommeD46/+^*, and between male *Nrf1^MommeD46/+^* and female *Nrf1^MommeD46/+^*. The number of litters analyzed for each intercross are indicated. (**d**) Table showing the number of pups obtained with genotypes *Nrf1^+/+^* and *Nrf1^MommeD46/+^* from the intercrosses between male *Nrf1^MommeD46/+^* and female *Nrf1^+/+^* and between male *Nrf1^+/+^* and female *Nrf1^MommeD46/+^*. The respective percentages appear in parenthesis. (**e**) Whisker plots indicating the relative weight ± SD of *Nrf1^+/+^* and *Nrf1^MommeD46/+^* mice at 3 weeks after birth, for each sex. (**f**) Representative images *Nrf1^+/−^*, *Nrf1^MommeD46/+^ and Nrf1^MommeD46/MommeD46^* embryos of 9.5 and 10.5 dpc. Scale bars indicate 1mm. ♂ and ♀ mean male and female, respectively. Statistical significance in (**a**,**d**) was determined by Chi–Square test and/or Fisher’s exact test, and in (**c**,**e**) by two-tailed Student’s t-test (for females) or Mann–Whitney U test (for males). Levene’s F-test determined significant differences on variances when *p* < 0.05. ns means not significant.

Herein, we wanted to determine whether the newly discovered mutation in *Nrf1* was responsible for any phenotypic change or defects in mice. To do so, we performed different intercrosses between adult *Nrf1^MommeD46^*^/+^ mice. We observed proportions of *Nrf1*^+/+^, *Nrf1^MommeD46^*^/+^ and *Nrf1^MommeD46^*^/*MommeD46*^ to be significantly different than that expected by Mendel at weaning (*p* < 0.001) (Figure 5a). Interestingly, neither homozygous mice were observed at weaning nor at the embryonic stage of 14.5 dpc indicating an earlier embryonic lethality of the mutation in homozygosis. In the earlier embryonic stages of 8.5, 9.5 and 10.5 dpc, homozygous embryos were observed at decreased ratios than expected (not significant). When further analyzing the homozygous embryos, we detected the presence of empty decidua in some of them, all presumably being *Nrf1^MommeD46^*^/*MommeD46*^, while the rest resembled embryos (Figure 5b). However, some of them looked abnormal. Male and female heterozygous mice showed similar fertility, producing an average of 7.9 ± 2.1 pups per litter when the heterozygous progenitor was a male, and 7.7 ± 2.5 pups when the heterozygous progenitor was a female. As expected, the heterozygous intercross produced less pups per litter, specifically 6.8 ± 1.3 (Figure 5c). To determine any possible progenitor effect in the hereditability of the mutation, we genotyped the offspring of two types of intercrosses: a heterozygous mutant male crossed with a wild-type female, and a wild-type male crossed with a heterozygous mutant female. The expected Mendelian proportions for both intercrosses were 50% wild-type and 50% heterozygous mutants. However, we observed different proportions in both intercrosses, with the ratios significantly different than expected (*p* < 0.001) for the intercross including a female wild-type. Moreover, the percentage of wild-type pups obtained from the intercross containing the female wild-type was significantly greater (75%) than the percentage obtained from the intercross having the male wild-type (62%) (*p* < 0.020) (Figure 5d). Altogether, these results suggest an accentuated maternal hereditability and some degree of haploinsufficiency of the described mutation in *Nrf1* which is further discussed in the next section. Finally, to ascertain any possible embryonic or adult defects caused by the mutation in *Nrf1*, we determined the body weights of *Nrf1*^+/+^ and *Nrf1^MommeD46^*^/+^ mice at weaning and explored the morphology of *Nrf1*^+/+^, *Nrf1^MommeD46^*^/+^ and *Nrf1^MommeD46^*^/*MommeD46*^ embryos at different embryonic stages. As seen in Figure 5e, heterozygous mutant mice weighed less than their wild-type counterparts in both sexes (not significant), with significantly different variances found in males (Levene’s F-test, *p* < 0.05). When examining the embryos at 8.5 and 9.5 dpc, we observed that homozygous mutant embryos were clearly smaller and less developed than the wild-type or heterozygous mutants (Figure 5f). However, no differences were detected between wild-type and heterozygous mutant embryos. This observation highlights the importance of Nrf1 for correct embryonic development.

## 4. Discussion

By deploying a forward dominant ENU mutagenesis screen in mice, we have discovered a novel missense mutation in *Nrf1* capable of altering the epigenetic state. Additional mutations were found in the link obtained by SNP-Chip but outside the fine mapped interval (Figure S4), which reassured us that the mutation in *Nrf1* was causative of the phenotype. Nrf1 is an enhancer of variegation as seen by the decrease in the percentage of GFP positive erythrocytes in heterozygous mutant mice. The exonic mutation in *Nrf1* is responsible for the amino acid (aa) change L ˃ P at the position 71 of the Nrf1 protein (total length: 503 aas) and is located in the aa region responsible for Nrf1 dimerization. This region ranges between 1 and 78 aas as the previously generated 1-to-78-deletion mutant has been shown to be entirely monomeric [22]. This same mutant also binds DNA less robustly compared to the wild-type protein [22], consistent with a possible overlap with the Nrf1 DNA binding domain, predicted to be between 79 and 172 in the chicken Nrf1 [23]. This suggests that the 71L ˃ P substitution of *MommeD46* could perturb both the homodimerization and DNA binding ability of *Nrf1*. *Nrf1^MommeD46^*^/+^ and *Nrf1^MommeD46^*^/*MommeD46*^ embryos show substantial reduction in the *Nrf1* mRNA levels indicating that the mutation could affect mRNA synthesis and processing. In contrast, Nrf1 protein levels do not seem to be altered in the mutant embryos. This could be due to increases in the half-life of the mutated Nrf1 protein or an increase in the *Nrf1^MommeD46^* mRNA translation, processes that have been reviewed in Liu et al. [24]. As for studies with *Nrf1*, Dhar et al. found a drastic reduction in *Nrf1* mRNA levels by qRT-PCR accompanied with a not so accentuated decrease in protein levels by Western blot in murine neuroblastoma cells [25].

We are the first to demonstrate a role of Nrf1 in maintaining DNA hypomethylation in vivo as seen by the significant hypermethylation of the α-globin HS-40 enhancer region observed in *Nrf1^MommeD46^*^/+^ and *Nrf1^MommeD46^*^/*MommeD46*^ embryos in comparison with wild-type embryos. Interestingly, only this present *MommeD46* and the previously published *MommeD28* (Rlf null mutant) [7] show hypermethylation in the HS-40 enhancer. This is consistent with the decreased expression of the GFP transgene. Although the preference of NRF1 to bind un-methylated regions [26,27] and methylated sequences [28] has been studied in depth, little is known about the role of Nrf1 in directly controlling DNA methylation. In this line, one study has found that Nrf1 transcriptionally activates Euchromatic histone methyltransferase 1 (EHMT1) during in vitro meiosis, and that its DNA binding ability is negatively regulated by CDK2-mediated phosphorylation [29]. However, the authors did not assess DNA methylation changes. In another work, the authors did not find changes in DNA methylation in the gene promoters of the six Nrf1 target genes analyzed in CD9+ spermatogonia from *Nrf1* germ cell-conditional knockout (*Nrf1*^f/f^) mice [27]. In contrast, in our study, Nrf1 was isolated from a genome-wide genetic screen in search of epigenetic modifiers and we found methylation changes in the HS-40 enhancer in mutants with dysfunctional Nrf1. Besides all this, it could be that NRF1 indirectly maintains DNA hypomethylation. The mutation L71P in Nrf1 could reduce the ability of Nrf1 to bind the DNA, such that Nrf1 is then unable to protect the CpG sites from methylation, leading to a methylation gain as previously described [26].

According to our embryonic and adult examinations of *MommeD46*, *Nrf1^MommeD46/MommeD46^* mice are not viable at birth and die before 14.5 dpc. Also, the offspring of the heterozygous intercross present significantly altered Mendelian ratios at weaning. Similarly, the *Nrf1* full knockout has shown to be embryonic lethal between 3.5 and 6.5 dpc [30]. Regarding mice fertility, our mutation in *Nrf1* does not seem to affect fertility as seen by the similar average litter sizes obtained from the cross *Nrf1*^+/+^ × *Nrf1^MommeD46^*^/+^ for both progenitor genders. However, in the literature, conditional ablation of Nrf1 in gonocytes has been shown to lead to infertility in male mice [27]. This contrasting evidence could be due to the subtle nonobvious effect that our mutation has on fertility. We observed that the wild-type *Nrf1* allele is significantly more present than expected in the offspring when the progenitor is a wild-type female rather than when it is a wild-type male (75% versus 62%). This could be explained by paternal imprinting of the mutated allele in the mating ♀*Nrf1*^+/+^ × ♂*Nrf1^MommeD46^*^/+^, by maternal imprinting of the mutated allele in the mating ♀*Nrf1^MommeD46^*^/+^ × ♂*Nrf1*^+/+^, by haploinsufficiency or by the existence of an additional ENU mutation having a cis- or trans-acting effect on the wild-type or the mutated allele. Maternal imprinting or inhibitory cis-/trans-acting effects on the mutated allele does not occur because the proportion of *Nrf1^MommeD46^*^/+^ progeny from the mating ♀*Nrf1^MommeD46^*^/+^ × ♂*Nrf1*^+/+^ is not significantly different from the expected Mendelian ratios, 38% versus 50%. In contrast, the possibility of activating cis- or trans-activating effects on the wildtype allele due to another ENU mutation cannot be ruled out, but, at the same time, is very difficult to prove. Regarding *Nrf1* inheritance, the literature shows that there is no maternal inheritance of *Nrf1* gene products as wild-type embryo eggs (*Nrf1*^+^) from the *Nrf1*^null/+^ heterozygous intercross were seen at the stage before the first meiotic division [30]. Regarding paternal imprinting, NRF1 has previously been shown to positively regulate the small nuclear ribonucleoprotein polypeptide N (SNRPN) locus which is associated with the imprinting center that regulates the Prader–Willi syndrome domain (PWS-IC), suggesting a relevant contribution of NRF1 in PWS-IC activity [31,32]. In other words, NRF1 orchestrates the characteristic loss of expression of the paternally-inherited allele of an imprinted gene cluster, that includes SNRPN, in the PWS. This suggests that the mutation in *MommeD46*, which affects the dimerization and DNA binding capabilities of Nrf1, could generate imprinting defects and be responsible for the increase in the expression of the maternal wild-type allele observed. Another possibility that could explain the greater presence of the wild-type allele in the offspring when the progenitor is a wild-type female is the haploinsufficiency caused by the mutated *Nrf1* allele in the heterozygous mice. The early lethality of the homozygous mutants supports this notion.

Regarding the effects of the mutation in embryos and adult phenotypes, we observed a decrease in size and developmental delay in 9.5 and 10.5 *Nrf1^MommeD46/MommeD46^* embryos compared to wild-type embryos. Moreover, both male and female *Nrf1^MommeD46/+^* mice display a slight decrease in body weight compared to wild-type mice at weaning, with significantly different variances in males. Similarly, a previous study demonstrated defective development in vitro of all *Nrf1*-null and some *Nrf1* heterozygous blastocysts as opposed to their wild-type counterparts [30]. In mice, *Nrf1* is highly expressed in thymocytes, B-cells and retina, and in humans, in natural killer cells, T cells and skeletal muscle. Consistent with that, retinal progenitor cell and postmitotic rod photoreceptor cell-specific *Nrf1* conditional knockout mice exhibit rod photoreceptor cell degeneration as a result of mitochondrial impairment [33]. In addition, Drosophila erect wing (ewg) (*Nrf1* homologue) mutants show aberrant axonal projection and absent or reduced indirect flight muscles [34], and zebrafish nrf (*Nrf1* homologue) mutants exhibit loss of retinal photoreceptors and reduced brain size [35]. Also in the neural context, reduced transcriptional activity of NRF1 has been associated with fragile X mental retardation syndrome [36] and with GABA-associated neuronal disorders [37], and functional NRF1 has been shown to be necessary for neurite outgrowth of human neuroblastoma cells [38,39]. Other non-neuronal roles attributed to NRF1 are the regulation of oncogenesis [40,41,42,43], stem cell aging [44], telomere transcription [45] and oxidative stress protection and lipid homeostasis in the liver; liver-specific *Nrf1* conditional knockout mice develop steatohepatitis and spontaneous liver cancer [43]. In addition, low levels of (mRNA) *Nrf1* have been observed in Cystathionine β synthase (CBS) heterozygous-null mice modeling hyperhomocysteinemia, a syndrome characterized by skeletal muscle weakness and fatigability [46]. However, it remains unknown whether Nrf1 is a causative factor or a consequence of the disease. Interestingly, CBS+/− mice exhibit significant weight loss [47], and homocysteine treatment, as a way to model the disease in vitro, increases the expression of DNA methyltransferase (DNMT)3a and DNMT3b and consequent global methylation levels in muscle C2C12 cells [46], which is in agreement with our findings of Nrf1 controlling the epigenetic state and the decrease in body weight observed in *Nrf1^MommeD46/+^* mice. Alternatively, given that Nrf1 controls muscle energy production [48], it could be that the decrease in body weight in the heterozygous *Nrf1* mutants is due to the reduction in muscle function and subsequent muscle mass. Further investigations will be undertaken to unravel other phenotypic traits or defects caused by the 71L ˃ P substitution in Nrf1.

## 5. Conclusions

By using a genome-wide mutagenesis screen, we have identified Nrf1 acting as a controller of the epigenetic landscape in mammals, through the maintenance of DNA hypomethylation in vivo. This novel *MommeD46* causes a reduction in the *Nrf1* mRNA levels and severe embryonic developmental defects. Additionally, we noted a peculiar hereditability of the mutation which is consistent with a paternal imprinting of the *Nrf1^MommeD46^* allele and haploinsufficiency. Our study unveils an important new epi-role of NRF1 in addition to its extensive transcriptional regulation throughout the animal life. Furthermore, the variant mouse produced in our laboratory could be used to model or elucidate unknown syndromes driven by a reduced function of NRF1.

## Supplementary Materials

The following are available online at https://www.mdpi.com/article/10.3390/ani11072103/s1, Figure S1: fine mapping of *MommeD46*, Figure S2: exome sequencing in *MommeD46*, Figure S3: Full Western Blot images and band quantification corresponding to Figure 3d. Full Western Blot images of (a) Nrf1 and (b) γ-Tubulin, Figure S4: Deep genome sequencing of the linked interval in *MommeD46*.
