# Supplementary material for: An N-ethyl-N-Nitrosourea Mutagenesis Screen in Mice Reveals a Mutation in Nuclear Respiratory Factor 1 (Nrf1) Altering the DNA Methylation State and Correct Embryonic Development
Source: Animals (Basel). 2021 Jul 15;11(7):2103. doi: 10.3390/ani11072103 (PMC8300126; doi:10.3390/ani11072103)
Supplement: Supplementary file 1 [file animals-11-02103-s001.zip › animals-1253949-supplementary.pdf]

Oligonucleotides used for fine mapping of *MommeD46*

Name ID	Name	Position	Forward oligonucleotide	Reverse oligonucleotide	Product size (bp)
1	*D6Mit38*	4,503,823 - 4,503,937	5'-GCTCTTATTAATGAAGAAGAA-3'	5'-CAAAGAAAGCATTTCAAGACT-3'	115
2	*rs13478656*	21,893,684 - 21,894,134	5'-CACCTTCAAAATTCCCCTGT-3'	5'-TCGTTTTGGCATCTGGTGTA-3'	450
3	*D6Mit268*	34,684,663 - 34,684,784	5'-TTTAAACTCCCAATAAAAAAC-3'	5'-CAGCCAACAAAAATAAAACCA-3'	122
4	*rs30220430*	38,073,424 - 38,073,851	5'-TTGCAGTCTTGATGCCACA-3'	5'-TCATGCCCACCAGATATCAA-3'	427
5	*rs6264329*	41,444,463 - 41,444,924	5'-AATATATGGCCACTAGGTCAGAGG-3'	5'-GCTAATGGAAAGCCTGGTGAT-3'	461
6	*D6Mit274*	48,726,565 - 48,726,677	5'-TCCTTCTCCATTTACACTTAC-3'	5'-GCAATGCCAAAATGTTCAAAT-3'	113
7	*D6Mit188*	75,447,583 - 75,447,710	5'-CTTTAGTCATTATTAGGATTG-3'	5'-TGGGATAGCATTGGAAACGTA-3'	128
8	*D6Mit105*	107,795,610 - 107,795,846	5'-AGTGTCAACTAACCCGGACCC-3'	5'-ATCCCCCCTACCCTCTCCTCT-3'	237
9	*D6Mit52*	127,838,217 - 127,838,360	5'-AAGGCACCTATATTTGTGCAC-3'	5'-TAAGTCAGCCCAAGGAAGTCA-3'	144

Supplemental Figure S1. Fine mapping of *MommeD46*. Table containing the oligonucleotides used for performing fine genomic mapping in *MommeD46.* The standard name of the position, the genomic position, the sequence 5’-3’ and the product size of the amplicon are specified for each oligonucleotide pair.

Exome sequencing in *MommeD46*

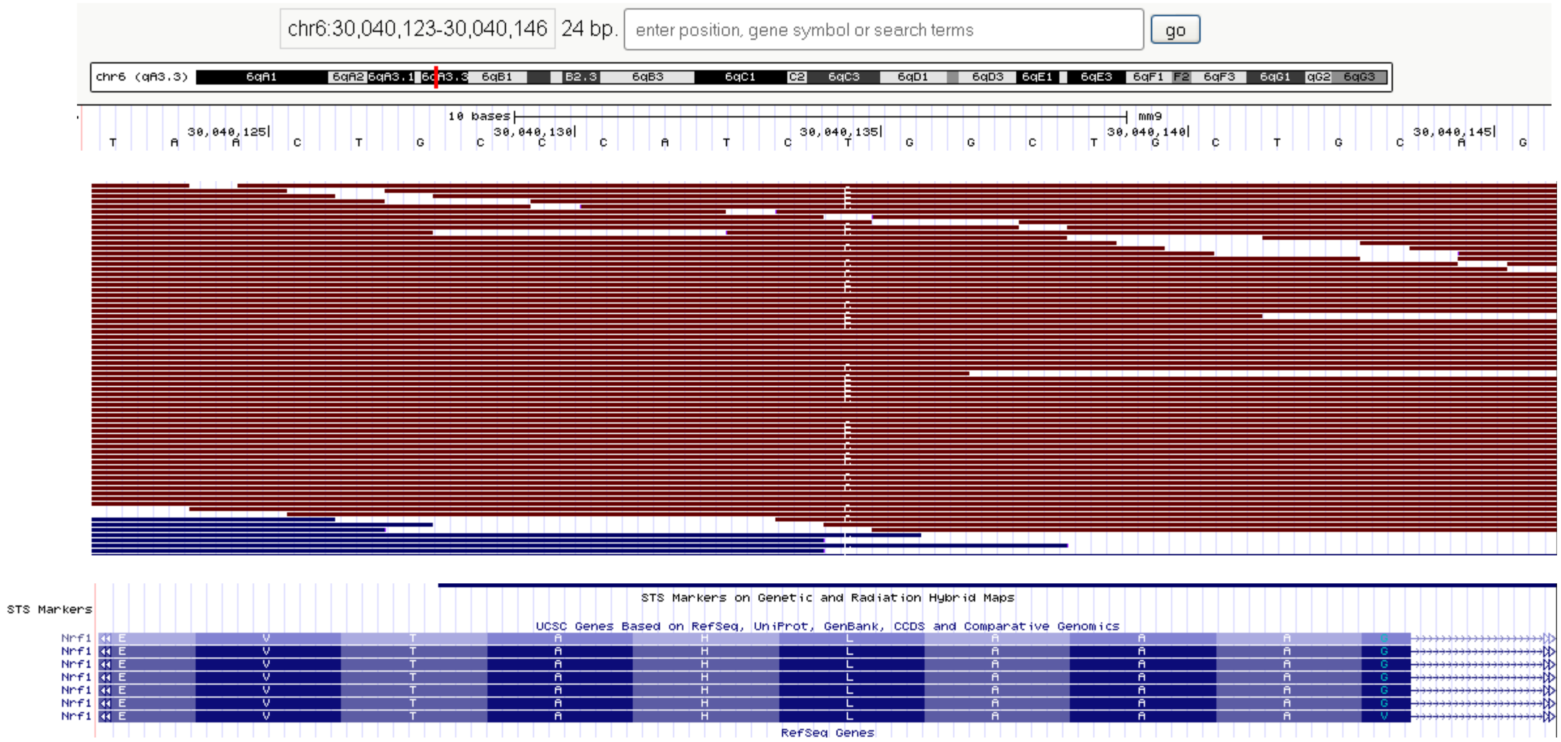

Supplemental Figure S2. Exome sequencing in *MommeD46*. Image of the sequence of the genomic region in Chr 6 carrying the causative mutation in *MommeD46*. A nucleotide substitution (T > C) in the position Chr6:30,040,135 can be seen in the genomic reads of the mouse variant.

Sorolla_Supplemental_Fig_S3

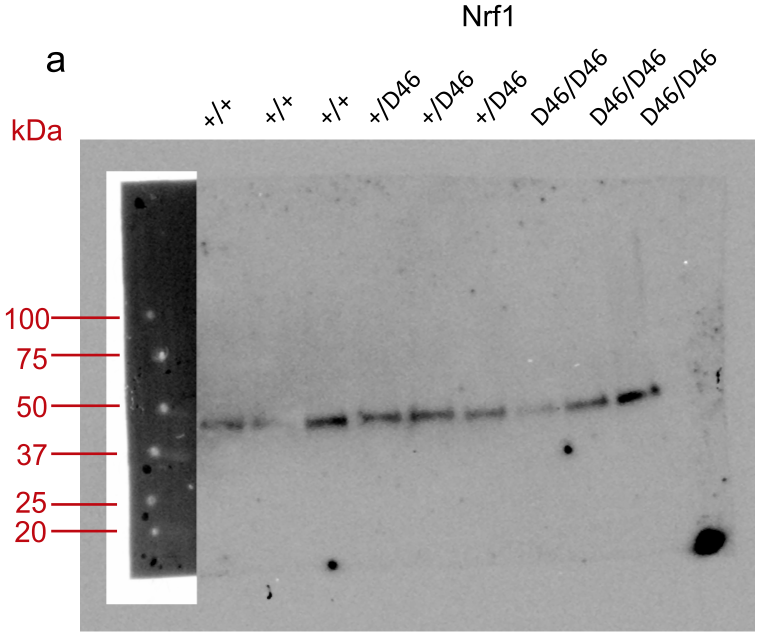

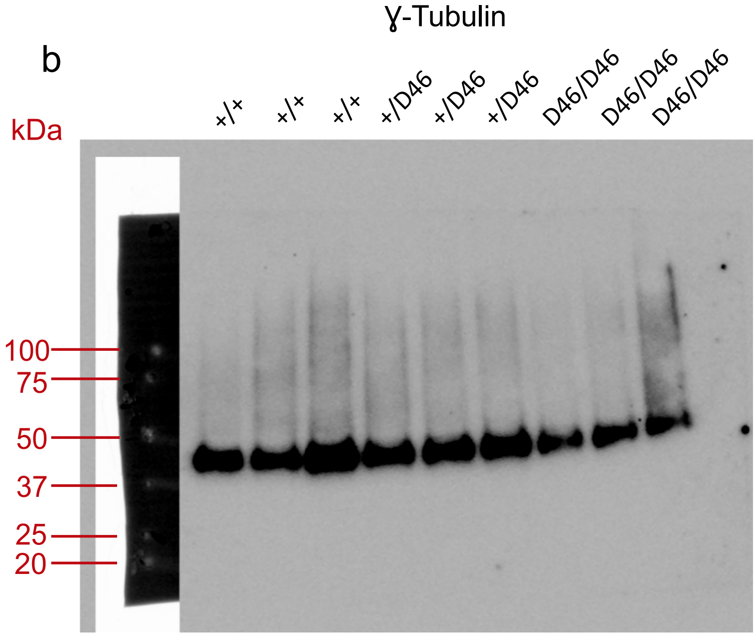

c

Condition	Total band volume: Nrf1	Total band volume: Ɣ-Tubulin	Total band volume normalized to Ɣ-Tubulin	Relative total band volume normalized to Ɣ-Tubulin
+/+	909266	2152645	0,42	1,00
+/+	878080	1962738	0,45	1,06
+/+	832736	3358258	0,25	0,59
+/D46	850725	2088352	0,41	0,96
+/D46	874722	2127664	0,41	0,97
+/D46	856295	2641740	0,32	0,77
D46/D46	786464	1649200	0,48	1,13
D46/D46	879138	1661880	0,53	1,25
D46/D46	1141440	1473725	0,77	1,83

ns

Supplemental Figure s3. Full Western Blot images and band quantification corresponding to Figure 3d. Full Western Blot images of (a) Nrf1 and

(**b**) **Ɣ-Tubulin.** (**c**)Table containing the total band volume of Nrf1 and Ɣ-Tubulin (intensity), the total normalized band volume of Nrf1 to Ɣ-Tubulin and the relative normalized total band volume of Nrf1 for the three different wild-type (+/+), heterozygous *MommeD46* (+/D46) and the homozygous *MommeD46* (D46/D46) embryos. Two-tailed Student's t-test was performed between the groups indicated. ns means not significant.

Genome sequencing in *MommeD46*

Region	Conversion	Codon change (Aa)	Exon/Intron	Gene
Chr6:38,275,413	T → A	N/A	Intron	*Zc3hav1*
Chr6:41,607,854	G → T	CTC (Leu) → CT**A** (Leu)	Exon	*Trpv5*
Chr6:48,600,373	T → C	GTA (Val) → G**C**A (Ala)	Exon	*Gimap8*

Supplemental Figure S4. Deep genome sequencing of the linked interval in *MommeD46*. Mutations found in the linked interval obtained by SNP-Chip
